# Transcriptional Analyses of Natural Leaf Senescence in Maize

**DOI:** 10.1371/journal.pone.0115617

**Published:** 2014-12-22

**Authors:** Wei Yang Zhang, Yong Chao Xu, Wen Lan Li, Long Yang, Xun Yue, Xian Sheng Zhang, Xiang Yu Zhao

**Affiliations:** 1 State Key Laboratory of Crop Biology, Shandong Key Laboratory of Crop Biology, College of Life Sciences, Shandong Agricultural University, Tai’an, Shandong, China; 2 College of Plant Protection, Shandong Agricultural University, Tai’an, Shandong, China; 3 College of Information Sciences and Engineering, Shandong Agricultural University, Tai’an, Shandong, China; Nanjing Agricultural University, China

## Abstract

Leaf senescence is an important biological process that contributes to grain yield in crops. To study the molecular mechanisms underlying natural leaf senescence, we harvested three different developmental ear leaves of maize, mature leaves (ML), early senescent leaves (ESL), and later senescent leaves (LSL), and analyzed transcriptional changes using RNA-sequencing. Three sets of data, ESL vs. ML, LSL vs. ML, and LSL vs. ESL, were compared, respectively. In total, 4,552 genes were identified as differentially expressed. Functional classification placed these genes into 18 categories including protein metabolism, transporters, and signal transduction. At the early stage of leaf senescence, genes involved in aromatic amino acids (AAAs) biosynthetic process and transport, cellular polysaccharide biosynthetic process, and the cell wall macromolecule catabolic process, were up-regulated. Whereas, genes involved in amino acid metabolism, transport, apoptosis, and response to stimulus were up-regulated at the late stage of leaf senescence. Further analyses reveals that the transport-related genes at the early stage of leaf senescence potentially take part in enzyme and amino acid transport and the genes upregulated at the late stage are involved in sugar transport, indicating nutrient recycling mainly takes place at the late stage of leaf senescence. Comparison between the data of natural leaf senescence in this study and previously reported data for Arabidopsis implies that the mechanisms of leaf senescence in maize are basically similar to those in *Arabidopsis*. A comparison of natural and induced leaf senescence in maize was performed. Athough many basic biological processes involved in senescence occur in both types of leaf senescence, 78.07% of differentially expressed genes in natural leaf senescence were not identifiable in induced leaf senescence, suggesting that differences in gene regulatory network may exist between these two leaf senescence programs. Thus, this study provides important information for understanding the mechanism of leaf senescence in maize.

## Introduction

During the life-cycle, a leaf undergoes at least three different development phases: a functional increasing phase at the early growth stage; a full functionality phase at the mature stage; and a functionality decreasing phase at the senescence stage [1]. Although leaf senescence is regarded as a massive operation of programmed cell death, it contributes critically to plant fitness by controlling the remobilization of micro- and macro-nutrients to growing and reproductive organs. It is a complex developmental process that is controlled by interactions of various internal (e.g., age and hormones) and environmental factors (e.g., drought and UV-B irradiation) [2], [3]. During senescence, numerous changes occur at the organic, cellular, and molecular levels in a highly synchronized manner [4].

Previous studies have elucidated the molecular mechanisms underlying leaf senescence through identification and characterization of senescence-associated genes (SAGs) and senescence-related mutants in plants such as *Arabidopsis* [5]–[15]. Genetic and genome-wide analyses suggest that the regulation of gene expression is complex during leaf senescence, with a large number of genes exhibiting differential expression patterns during senescence. At the onset of leaf senescence, a subset of SAGs is up-regulated and the majority of genes expressed in non-senescent leaves are down-regulated, including photosynthesis-related genes.

Transcription factors (TFs) mediate gene expression by activating and suppressing the expression of target genes [16], [17]. They play important roles in the regulation of leaf senescence in *Arabidopsis*. The largest groups of senescence-related TFs include members of the NAM, ATAF, CUC (NAC), WRKY, MYB, C2H2 zinc-finger, bZIP, APETALA2, and ethylene-responsive element binding protein (AP2/EREBP) families [55]. Among these, a number of WRKY TFs *WRKY6*, *WRKY18*, *WRKY22*, *WRKY29*, *WRKY53*, *WRKY54*, and *WRKY70* are involved in the regulation of plant defense and senescence in *Arabidopsis* [9], [18]–[22]. Several members of the NAC family of TFs function in regulating *Arabidopsis* senescence [23]. Mutants of *NAC092/AtNAC2/ORESARA1* (*ORE1*), which encodes a senescence-promoting regulator, delay leaf senescence [24]. Seventy-eight SAGs from 170 genes downstream of *ORE1* were significantly up-regulated in *ORE1*-overexpressing plants [25]. Of these, *BIFUNCTIONAL NUCLEASE1* (*BFN1*), *SINA1*, and *SAG29/SWEET15* are direct targets of ORE1 [26]. Some NAC genes are up-regulated by ORE1 during leaf senescence [8]. For example, NAP and ORS1 have been characterized as senescence-promoting regulators. Their mutants exhibit delayed leaf senescence, and plants overexpressing these genes display early leaf yellowing during leaf senescence [15], [23], [27]. *VND-INTERACTING2* (*VNI2*) encodes a NAC transcription factor and serves as a molecular integrator between abscisic acid signals and leaf senescence [28]. Leaf aging was delayed in *VNI2*-overexpressing plants and accelerated significantly in *vni2-1* mutant. Other TFs, such as AtARF2, AtARR2, AtMYB2, and MtATB2 have also been shown to be involved in plant senescence [29]–[32]. Therefore, TFs play essential roles in the fine-tuning of the senescence of plants. However, their biological function in senescence remains to be investigated.

A typical feature of plant senescence is the ordered degradation of macromolecules and the redistribution of products in plant tissues. Transporters are the elementary carriers of substances in plants, and they play an important role in various plant developmental processes, including leaf senescence. In a large-scale microarray study, 74 putative transporter (TPs) genes showed increased expression during developmental senescence in *Arabidopsis* [6]. During natural leaf senescence, the expression of 153 TPs genes was enhanced [7]. Up-regulation of amino acid and oligopeptide TPs correlates with extensive protein degradation during senescence, and they export breakdown products to the sink organs [33]. In crops, the remobilization of nutrients from vegetative parts to reproductive structures during leaf senescence can influence crop productivity [34], and TPs are involved in the process of nutrient remobilization. Further elucidation and characterization of the molecular functions of TPs involved in plant senescence is likely to provide important information for crop genetic improvements.

As a powerful tool in plant biology studies, high-throughput gene expression analysis has allowed for the investigation of molecular mechanisms underlying leaf senescence on a whole-genome basis [5]–[8], [10], [35]. In addition to developmental regulation, leaf senescence can be induced by various stresses including darkening and starvation. Comparative transcriptome analysis demonstrated that gene expression patterns and signalling pathways in natural leaf senescence are significantly different from those in induced leaf senescence [6], [7].

Maize is an important crop that undergoes whole plant senescence to maximize energy input into reproductive structures, which has a direct affect upon productivity. Understanding the underlying molecular mechanisms of maize leaf senescence will be helpful for improvements in yield. Transcriptional analysis has previously been performed during induced senescence by preventing pollination in maize [10]. However, the transcriptional profile in the natural leaf senescence of maize remains to be investigated. In this study, the global gene expression profile of the leaf at three important developmental stages during natural leaf senescence was investigated through RNA sequencing (RNA-Seq) analysis. In total, 4,552 differentially expressed genes were identified, including both well-known and candidate genes involved in the natural leaf senescence of maize. Gene ontology (GO) analysis revealed that the enrichment of genes differentially expressed during maize leaf senescence was focused on 12 biological pathways. A comparison of transcriptional data was conducted between maize and *Arabidopsis* [5], and between natural and induced leaf senescence [10] in maize. These data further our understanding of the regulation mechanisms underlying leaf senescence in maize.

## Results and Discussion

### RNA-Seq analysis of natural leaf senescence at three developmental stages

**Figure 1:**
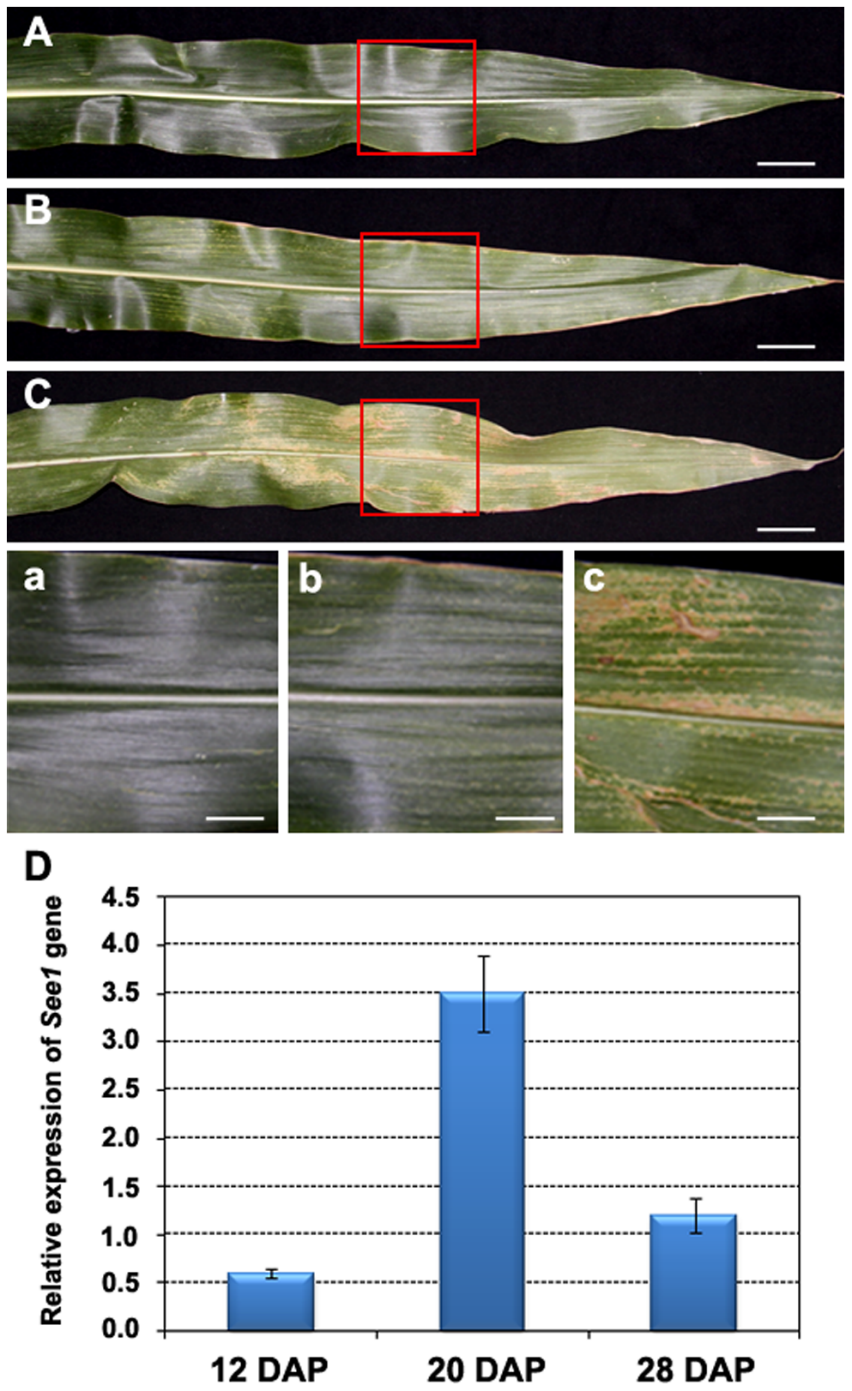
Morphology and molecular identification of leaf tissues for RNA-sequencing. (A–C) Maize ear leaves at three different developmental stages: (A) 12 DAP, (B) 20 DAP, and (C) 28 DAP. Picture in (a), (b), and (c) is close-up views of the region labelled in the red box in A–C, respectively. (D) *See1* gene expression patterns in leaves at three different developmental stages. Scale bars  =  100 mm (A–C), 1 cm (a to c).

Generally, a leaf goes through three main development phases: an expansion phase, a maturity phase, and a senescence phase [36]. The second phase, the time from the maturity of a leaf to the onset of leaf senescence is considered the functional phase attributing to crop yield. The ear leaf, the largest leaf in maize plant, is thought to play an important role in nutrient recycling to the cob during leaf senescence [37]. The timing of maize leaf senescence various depends on the varieties [37]. The chlorophyll content and photosynthesis capacity in the ear leaf of inbred Q319 will declines approximately two weeks after pollen shed [38]. According to the physiological change of the ear leaf, the developing ear leaf after pollination can be divided into three classes: mature leaves [ML, 0–14 days after pollination (DAP)]; early senescent leaves (ESL, 15–24 DAP); and later senescent leaves (LSL, 25–30 DAP). In ML, the chlorophyll content in the ear leaf remains at the maximum level, and the photosynthesis rate reaches its peak value. In ESL, the chlorophyll content begins to decrease, and the photosynthesis rate slows. In LSL, complexes of chlorophyll and proteins are degraded, and the photosynthetic capacity of the leaf declines sharply. To confirm these three leaf classes, we harvested ear leaves at 12 DAP, 20 DAP, and 28 DAP, representing ML, ESL, and LSL, respectively (Fig. 1A–C), and determined the expression of *See1* [37], [39], a maize ortholog of the *Arabidopsis* senescence marker gene *SAG12* [18], [40], [41]. In leaves, *See1* was expressed at a low level at 12 DAP, while its expression was up-regulated approximately 6-fold at 20 DAP (Fig. 1D). Expression then fell to a very low level at 28 DAP (Fig. 1D). Therefore, these samples met the criterion for the leaf senescence process and could be used in this study.

**Figure 2:**
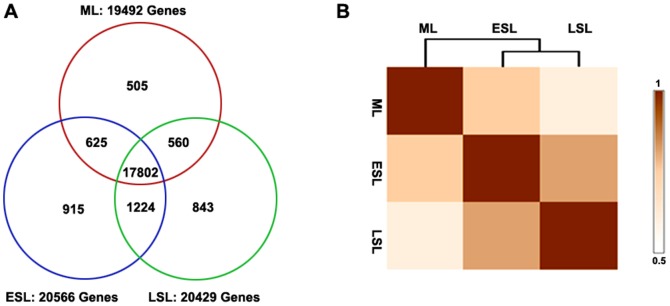
Gene expression of maize leaves during natural senescence and correlation matrices of their RNA-seq libraries. (A) Distribution of genes expressed in the three studied maize leaves. (B) Spearman correlation coefficient analysis of the RNA-seq libraries.

To identify genes involved in the leaf senescence process, we sequenced three cDNA libraries, ML (12 DAP), ESL (20 DAP), and LSL (28 DAP) using an Illumina HiSeqTM 2000. After removal of dirty raw reads, the number of filtered clean reads in each library was 11,914,022, 12,381,189, and 11,788,942, respectively (S1 Table), a tag density deemed sufficient for quantitative analysis of gene expression. To determine the genes corresponding to the reads in the three libraries, the filtered clean reads were mapped to version 2 of the maize B73 reference genome (AGPv2) [42] using the Short Oligo-nucleotide Alignment Program 2 (SOAP2) aligner [43]. To ensure that the libraries were meaningful, reads that appeared only once were eliminated from further statistical analysis. The analysis was extended to investigate global patterns of gene expression during the three developmental stages of leaf senescence to identify common and different characteristics. Both unique and overlapping genes were detected in the three samples. In all, 19,492 (ML), 20,566 (ESL), and 20,429 (LSL) genes were detected (Fig. 2A, S2 Table). A total of 17,802 genes (91.33% of expressed genes in ML, 86.56% of expressed genes in ESL, and 87.14% of expressed genes in LSL) were constitutively expressed at the three stages (Fig. 2A).

To establish relationships between the experimental samples, a Pearson correlation coefficient (PCC) analysis was performed on the sequencing libraries of the three samples. The gene expression profiles in ESL and LSL showed higher similarities (PCC - ESL/LSL  =  0.817) than those of ML and ESL (PCC - ML/ESL  =  0.756) (Fig. 2B).

### Changes in gene expression profiles in maize senescent leaf

To screen for genes differentially expressed during the natural senescence of maize leaves, significance of digital gene expression analysis was performed [44]. Three sets of data were compared: ESL vs. ML, LSL vs. ML, and LSL vs. ESL. All genes with different expression in three defined samples were defined as the differentially expressed genes (DEGs) during the leaf senescence. With the filter criteria of fold change ≥ 2.0 and false discovery rate (FDR) ≤0.001, 2,771, 2,082, and 3,117 DEGs were identified derived from the three selected comparisons, respectively (S3 Table). In the first comparison (ESL/ML), the expression of 1,773 genes was up-regulated, whereas 998 genes were down-regulated (S3A Table). In the second comparison (LSL/ML), the expression of 1,144 genes were increased and 945 genes were declined (S3B Table). In the third comparison (LSL/ESL), 1,999 genes had peak expression in LSL, and 1,118 genes had peak expression in ESL (S3C Table).

**Figure 3:**
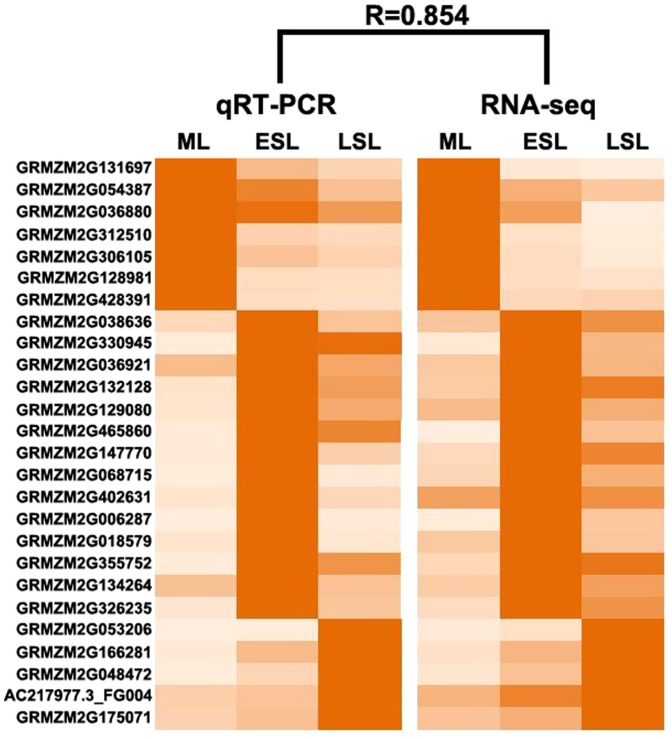
Validation of RNA-seq results by RT-qPCR. Expression levels of 26 randomly selected genes from the three studied samples as detected by qRT-PCR. Expression levels represented by the two heat maps are the average of gene expression values from three independent real-time PCR experiments (left), and normalized RPKM values of RNA-seq analysis (middle). For each gene, the tissue with the maximum expression level was regarded as 100, and relative expression levels of the other three tissues were calculated according to this maximum level. Relative expression is represented by colour scales as indicated (right). R is the correlation coefficient value between the two platforms.

To determine the accuracy of the RNA-seq data, quantitative reverse-transcription PCR (RT-qPCR) analysis of the differentially expressed genes was conducted to verify the expression patterns in the three samples. The expression of 26 randomly selected genes (S4 Table), including 15 genes with function annotation, eight genes encoding putative proteins, and three unknown genes, was analyzed by qRT-PCR. The expression patterns obtained by qRT-PCR strongly correlated with the RNA-seq results (R = 0.854), indicating that the RNA-seq data was reliable (Fig. 3).

**Figure 4:**
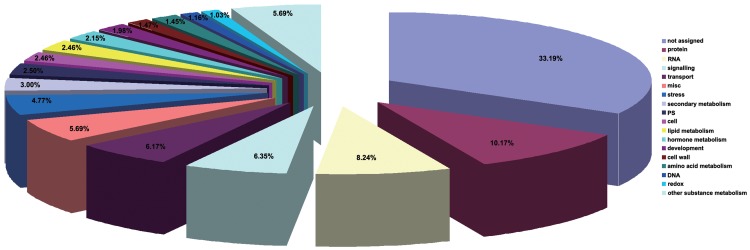
Classification of differentially expressed genes in maize leaves during natural senescence. Classification results were obtained using the MapMan approach.

To understand the functions of genes with changed expression during leaf senescence, genes were classified into 18 functional categories in accordance with MapMan annotation [45]. As shown in Fig. 4, the largest category was unknown genes (33.19%). Most well-annotated genes were involved in protein (10.17%), RNA (8.24%), miscellaneous (6.35%), transport (6.17%), signalling (5.69%), other substance metabolism (5.69%), stress (4.77%), secondary metabolism (3.05%), photosynthesis (2.50%), cell (2.46%), lipid metabolism (2.46%), hormone metabolism (2.15%), development (1.98%), cell wall (1.47%), amino acid metabolism (1.45%), DNA (1.16%), and redox (1.03%) processes.

Chlorophyll content is a well-established senescence marker, with its degradation an integral part of the senescence syndrome. With the decline of the chlorophyll content, the photosynthetic rate is also markedly decreased during the senescence process. Leaf senescence is usually accompanied by decreased expression of genes involved in photosynthesis. In this study, photosynthetic genes were the most important group, and they were markedly down-regulated during leaf senescence, especially in the later stage (from 20 DAP to 28 DAP) (S3 Table). The expression of *GRMZM2G094224*, a *pasL* (At4g12800) homologue gene encoding subunit L of the photosystem I reaction center in *Arabidopsis* [46], was significantly down-regulated (log_2_Ratio  =  −16.818) and was barely detectable in LSL. In the comparison between LSL and ESL, 50 of the 2,084 differently expressed genes were down-regulated significantly, and ten genes were up-regulated (Table 1). Further analysis revealed that five up-regulated genes were involved in carbon metabolism that is specific to C_4_ plant photosynthesis (Table 1). It is likely that up-regulation of these genes provides essential energy for the maintenance of normal metabolism in maize during late senescence stage, even though the photosynthetic capacity was reduced at this stage.

**Table 1:** The differentially expressed photosynthesis associated genes in maize leaf during natural senescence.

GeneID	log_2_^(LSL/ESL)^	Gene Expression Change	Annotations
GRMZM2G089365	1.81	up-regulated	PS.calvin cycle.aldolase
AC203966.5_FG002	3.89	up-regulated	PS.lightreaction.cyclic electron flow-chlororespiration
GRMZM2G320305	2.11	up-regulated	PS.lightreaction.other electron carrier (ox/red).ferredoxin reductase
GRMZM2G159587	1.51	up-regulated	PS.photorespiration.hydroxypyruvate reductase
GRMZM2G358153	4.26	up-regulated	PS, calvin cycle, rubisco interacting
GRMZM2G066413^a^	1.43	up-regulated	PS, carbon concentrating mechanism, C4 (phosphate/Phosphoenolpyruvate translocator)
GRMZM2G069542^a^	1.08	up-regulated	PS, carbon concentrating mechanism, C4 (phosphoenolpyruvate carboxylase)
GRMZM2G085019^a^	1.32	up-regulated	PS, carbon concentrating mechanism, C4 (NADP-malic enzyme)
GRMZM2G118770^a^	1.72	up-regulated	PS, carbon concentrating mechanism, C4 (NADP-malic enzyme)
GRMZM2G348512^a^	2.77	up-regulated	PS, carbon concentrating mechanism, C4 (Carbonic Anhydrase)
GRMZM2G155253	−1.16	down-regulated	PS.calvin cycle.aldolase
GRMZM2G306732	−1.54	down-regulated	PS.calvin cycle.FBPase
GRMZM2G089136	−1.44	down-regulated	PS.calvin cycle.phosphoglycerate kinase
GRMZM2G450762	−1.57	down-regulated	PS.calvin cycle.rubisco large subunit
GRMZM2G179146	−1.23	down-regulated	PS.lightreaction.ATP synthase
GRMZM2G448142	−1.23	down-regulated	PS.lightreaction.ATP synthase.beta subunit
GRMZM2G025171	−1.43	down-regulated	PS.lightreaction.ATP synthase.delta chain
GRMZM2G391831	−2.24	down-regulated	PS.lightreaction.ATP synthase.subunit C
GRMZM2G075958	−1.02	down-regulated	PS.lightreaction.cyclic electron flow-chlororespiration
GRMZM2G102349	−1.12	down-regulated	PS.lightreaction.cyclic electron flow-chlororespiration
GRMZM2G086763	−1.26	down-regulated	PS.lightreaction.cyclic electron flow-chlororespiration
GRMZM2G012119	−1.95	down-regulated	PS.lightreaction.cyclic electron flow-chlororespiration
GRMZM2G167766	−1.01	down-regulated	PS.lightreaction.cytochrome b6/f
GRMZM2G162748	−1.21	down-regulated	PS.lightreaction.cytochrome b6/f
GRMZM2G038365	−1.38	down-regulated	PS.lightreaction.cytochrome b6/f
GRMZM2G463640	−1.21	down-regulated	PS.lightreaction.cytochrome b6/f.cytochrome b6 (CYB6)
GRMZM2G109244	−1.38	down-regulated	PS.lightreaction.NADH DH
GRMZM2G154667	−1.45	down-regulated	PS.lightreaction.NADH DH
GRMZM2G304947	−1.82	down-regulated	PS.lightreaction.NADH DH
GRMZM2G475437	−1.88	down-regulated	PS.lightreaction.NADH DH
GRMZM2G176129	−2.60	down-regulated	PS.lightreaction.NADH DH
GRMZM2G032253	−1.51	down-regulated	PS.lightreaction.other electron carrier (ox/red).ferredoxin
GRMZM2G122337	−1.95	down-regulated	PS.lightreaction.other electron carrier (ox/red).ferredoxin
GRMZM2G084279	−1.54	down-regulated	PS.lightreaction.other electron carrier (ox/red).ferredoxin oxireductase
GRMZM2G059191	−1.27	down-regulated	PS.lightreaction.other electron carrier (ox/red).ferredoxin reductase
GRMZM2G168143	−1.39	down-regulated	PS.lightreaction.other electron carrier (ox/red).ferredoxin reductase
GRMZM2G071450	−1.14	down-regulated	PS.lightreaction.other electron carrier (ox/red).plastocyanin
GRMZM2G016622	−1.03	down-regulated	PS.lightreaction.photosystem I.PSI polypeptide subunits
GRMZM2G001653	−1.11	down-regulated	PS.lightreaction.photosystem I.PSI polypeptide subunits
GRMZM2G085646	−1.15	down-regulated	PS.lightreaction.photosystem I.PSI polypeptide subunits
GRMZM2G017290	−1.37	down-regulated	PS.lightreaction.photosystem I.PSI polypeptide subunits
GRMZM2G012397	−1.46	down-regulated	PS.lightreaction.photosystem I.PSI polypeptide subunits
GRMZM2G024150	−1.85	down-regulated	PS.lightreaction.photosystem I.PSI polypeptide subunits
GRMZM2G057281	−1.04	down-regulated	PS.lightreaction.photosystem II.LHC-II
GRMZM2G033885	−1.88	down-regulated	PS.lightreaction.photosystem II.LHC-II
GRMZM2G132506	−1.05	down-regulated	PS.lightreaction.photosystem II.PSII polypeptide subunits
GRMZM2G101617	−1.11	down-regulated	PS.lightreaction.photosystem II.PSII polypeptide subunits
GRMZM2G308944	−1.13	down-regulated	PS.lightreaction.photosystem II.PSII polypeptide subunits
GRMZM2G436986	−1.15	down-regulated	PS.lightreaction.photosystem II.PSII polypeptide subunits
GRMZM2G176840	−1.24	down-regulated	PS.lightreaction.photosystem II.PSII polypeptide subunits
GRMZM2G067883	−1.53	down-regulated	PS.lightreaction.photosystem II.PSII polypeptide subunits
GRMZM2G394732	−1.54	down-regulated	PS.lightreaction.photosystem II.PSII polypeptide subunits
GRMZM2G134130	−1.88	down-regulated	PS.lightreaction.photosystem II.PSII polypeptide subunits
GRMZM2G166899	−3.09	down-regulated	PS.photorespiration.hydroxypyruvate reductase

Genes labelled “^a^” are specific to C_4_ plant photosynthesis.

**Figure 5:**
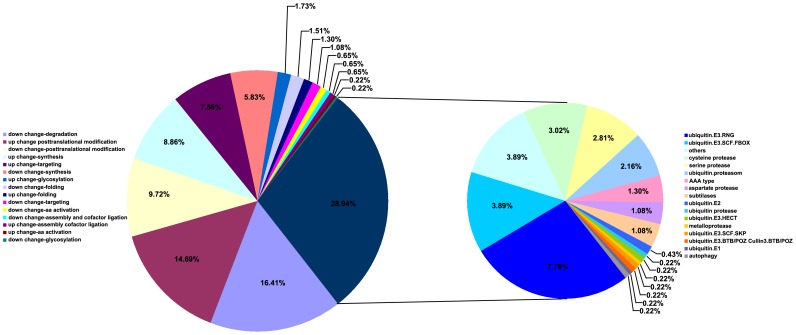
Distribution of protein metabolic related genes in early stage leaf senescence differentially expressed gene set. Protein metabolic related genes differentially expressed in early stage leaf senescence were classified into 16 categories by MapMan. Genes involved in protein degradation are shown to the right.

During plant development, leaf senescence is a pivotal process for the recycling of nutrients from senescing leaves to developing sinks, such as young leaves and seeds. In senescing leaf, complex macromolecules, including proteins, are broken down, and approximately two-thirds of the soluble proteins in plant cells are lost [2]. Therefore, protein degradation is an important symptom of senescence. Consistent with the decline of most proteins levels, representatives of the major classes of proteases (cysteine (Cys)-, aspartic-, serine-, and metallo-proteases) increased their activity or transcript expression levels [2]. In this study, genes involved in protein metabolism made up the largest group of differently expressed genes during leaf senescence; 45.5% of protein metabolism genes (210 genes) were related to protein degradation (Fig. 5). *SAG12* encodes a senescence-specific papain-like Cys-protease in *Arabidopsis* [47]. Its maize ortholog, *See1* (*GRMZM2G045706*), had an up-regulated transcript level during leaf senescence. Expression of *GRMZM2G048836*, which encodes a metallo-protease, was enhanced 8.23-fold during senescence. In *Arabidopsis*, its ortholog FtsH6 is involved in the degradation of Lhcb3 and Lhcb1 during senescence [48], [49].

Further analysis revealed that 60.07% of the up-regulated genes involved in protein degradation at earlier stages of leaf senescence (from 12 to 20 DAP) encode proteases in the ubiquitin-proteasome pathway (Fig. 5). Genetic studies revealed that this pathway performs an important regulatory role in leaf senescence [21], . We determined that the *GRMZM2G405203* gene was largely enhanced during the leaf senescence process. Its *Arabidopsis* ortholog, *ORE9,* encodes an F-box protein, a component of the SCF complex that acts as an E3 ligase in ubiquitin-dependent proteolysis; an *ore9* mutation delayed leaf senescence in *Arabidopsis* [53].

TFs activate/suppress target gene expression by binding to distinct cis-elements. These are generally located in the 5′ upstream regulatory regions of target genes. Among all genes annotated in the *Arabidopsis* nuclear genome [54], 185 TF genes were identified as being differentially regulated during leaf senescence [55]. The largest groups of senescence-regulated TFs were classified into the NAC, WRKY, MYB, C2H2 zinc-finger, bZIP, and AP2/EREBP families [55]. In maize, approximately 3355 TFs were identified and classified into 56 families [16], [17]. We identified 233 genes encoding transcription factors from the genes differentially expressed during maize leaf senescence. These were distributed into 35 subfamilies, including MYB, bHLH, C2H2, NAC, AP2/EREBP, and bZIP families (S5 Table). Many TFs involved in leaf senescence have been previously characterized [9], [11], [15], [25], [26−28], [32], . The WRKY and NAC subfamilies represent the largest groups and are well-documented [9], [15], [20], [23], [26], [28], . WRKY53 and ANAC092 (ORE1) act as positive regulators in *Arabidopsis* leaf senescence [20], [23], [24], [26], [62]. Expression of their maize orthologs was induced during leaf senescence (Table 2), suggesting that they also play important roles in the maize leaf senescence process.

**Table 2:** Genes show high similarity to well-known SAGs in the SDEG dataset.

GENE ID (Maize)	Homologous gene in Arabidopsis	Gene Description	Function Annotation	Reference(s)
AC234526.1_FG005	AT1G09500	cinnamyl-alcohol dehydrogenase family/CAD family (AtSAG26)	Up-regulated during developmental and induced senescence	[7], [82]
GRMZM2G032977	AT1G11190	BIFUNCTIONAL NUCLEASE I (BFN1)	Nucleic acid degradation during senescence	[6], [83]
GRMZM2G088212	AT1G20620	Catalase 3(CAT3), SENESCENCE 2	Induced by age and senescence	[84], [85]
GRMZM2G119168	AT1G20900	AHL27, AT-HOOK MOTIF NUCLEAR-LOCALIZED PROTEIN 27	Delayed leaf senescence	[86]
GRMZM2G047404	AT1G61800	GLUCOSE-6-PHOSPHATE/PHOSPHATE TRANSLOCATOR 2 (GPT2)	Senescence associated gene	[41], [87]
AC207347.3_FG005	AT1G73220	ORGANIC CATION/CARNITINE TRANSPORTER1 (OCT1)	Strong senescence up-regulation	[88]
GRMZM2G001645	AT1G76490	HYDROXY METHYLGLUTARYL COA REDUCTASE 1 (HMG1)	Negative regulator of leaf senescence	[89]
GRMZM2G106303	AT1G76680	12-OXOPHYTODIENOATE REDUCTASE 1 (OPR1)	Up-regulated by senescence and jasmonic acid	[90]
GRMZM2G125943	AT2G01830	CRE1/AHK4	Programmed cell death	[91]
GRMZM2G151223	AT2G01830	CRE1/AHK4	Programmed cell death	[91]
GRMZM2G074236	AT2G35980	YELLOW-LEAF-SPECIFIC GENE 9 (YLS9)	Induced during senescence.	[92]
GRMZM2G405203	AT2G42620	ORESARA 9 (ORE9)	Positive regulator of leaf senescence	[53]
GRMZM2G163251	AT2G43000	JUNGBRUNNEN 1 (JUB1), NAC DOMAIN CONTAINING PROTEIN 42 (NAC042)	Over expression of the gene strongly delays senescence.	[93]
GRMZM2G112548	AT2G43000	JUNGBRUNNEN 1 (JUB1), NAC DOMAIN CONTAINING PROTEIN 42 (NAC042)	Over expression of the gene strongly delays senescence.	[93]
GRMZM2G051943	AT2G43570	CHITINASE (CHI)	The senescence-enhanced genes	[94]
GRMZM2G007848	AT2G43790	MAP KINASE 6 (MPK6)	Positive regulator of leaf senescence	[95]
GRMZM2G084347	AT2G43790	MAP KINASE 6 (MPK6)	Positive regulator of leaf senescence	[95]
GRMZM2G140811	AT3G15730	phospholipase Dα1 (PLDα1)	Positive regulator in ABA-promoted senescence	[96]
GRMZM2G004694	AT3G18830	ARABIDOPSIS THALIANA POLYOL/MONOSACCHARIDE TRANSPORTER 5 (AtPLT5)	Abscission control	[97]
GRMZM2G062156	AT3G18830	ARABIDOPSIS THALIANA POLYOL/MONOSACCHARIDE TRANSPORTER 5 (AtPLT5)	Abscission control	[97]
GRMZM2G302604	AT3G18830	ARABIDOPSIS THALIANA POLYOL/MONOSACCHARIDE TRANSPORTER 5 (AtPLT5)	Abscission control	[97]
GRMZM2G063824	AT3G18830	ARABIDOPSIS THALIANA POLYOL/MONOSACCHARIDE TRANSPORTER 5 (AtPLT5)	Abscission control	[97]
GRMZM5G862325	AT3G18830	ARABIDOPSIS THALIANA POLYOL/MONOSACCHARIDE TRANSPORTER 5 (AtPLT5)	Abscission control	[97]
GRMZM2G339563	AT3G44880	PHEOPHORBIDE A OXYGENASE (PAO)	Positive regulator in leaf senescence	[98], [99]
GRMZM2G018484	AT3G44880	PHEOPHORBIDE A OXYGENASE (PAO)	Positive regulator in leaf senescence	[98], [99]
GRMZM2G152739	AT3G52430	PHYTOALEXIN DEFICIENT 4 (PAD4)	Involved in the GPA feeding-induced leaf senescence	[51], [100]
AC217401.3_FG001	AT3G60130	BETA GLUCOSIDASE 16, BGLU16	Leaf senescence associated genes	[7]
GRMZM2G108849	AT4G01610	AtCathB3	Up-regulated by dark-induced senescence.	[101]
GRMZM2G091837	AT4G22920	ATNYE1, NON-YELLOWING 1, NYE1, SGR, STAY-GREEN	Chlorophll degradation	[102], [103]
GRMZM2G030272	AT4G23810	WRKY53	Positive regulator of leaf senescence	[20], [62]
GRMZM2G060918	AT4G23810	WRKY53	Positive regulator of leaf senescence	[20], [62]
GRMZM2G103055	AT4G25000	ALPHA-AMYLASE-LIKE (AMY1)	AMY1 is involved in senescence-associated starch degradation.	[104]
GRMZM2G081626	AT4G32940	GAMMA VACUOLAR PROCESSING ENZYME (GAMMA-VPE)	Up-regulated during senescence.	[105]
GRMZM2G093032	AT4G32940	GAMMA VACUOLAR PROCESSING ENZYME (GAMMA-VPE)	Up-regulated in senescence	[105]
GRMZM2G000812	AT5G13170	SENESCENCE-ASSOCIATED GENE 29 (SAG29)	Leaf senescence associated genes	[106]
GRMZM2G324903	AT5G13170	SENESCENCE-ASSOCIATED GENE 29 (SAG29)	Leaf senescence associated gene	[106]
GRMZM2G109070	AT5G13800	CO-REGULATED WITH NYE1 (CRN1), PHEOPHYTINASE (PPH)	Chlorophyll breakdown	[107]
GRMZM2G048836	AT5G15250	FTSH PROTEASE 6 (FTSH6)	FtsH6 is involved in the degradation of both Lhcb3 and Lhcb1 during senescence and high-light acclimation.	[48], [49]
AC211394.4_FG004	AT5G34850	PURPLE ACID PHOSPHATASE 26 (PAP26)	Leaves of T-DNA insertion mutant displayed delayed senescence	[108]
GRMZM2G114850	AT5G39610	NAC DOMAIN CONTAINING PROTEIN 2 (ATNAC2), ANAC092, ORESARA1 (ORE1)	Positive regulator of leaf senescence	[23], [24], [26]
GRMZM2G327059	AT5G41410	BELL1	Positive regulator of leaf senescence	[109]
GRMZM2G144083	AT5G44790	RESPONSIVE-TO-ANTAGONIST 1 (RAN1)	ATP dependent copper transporter vital for ethylene response pathway	[110]
GRMZM2G045706	AT5G45890	SENESCENCE-ASSOCIATED GENE 12 (SAG12)	The senescence-specific gene	[18], [40], [41]
GRMZM2G172230	AT5G51070	SENESCENCE ASSOCIATED GENE 15 (SAG15), EARLY RESPONSIVE TO DEHYDRATION 1 (ERD1)	Leaf senescence associated genes	[40], [111], [112]
GRMZM2G040890	AT5G51070	SENESCENCE ASSOCIATED GENE 15 (SAG15), EARLY RESPONSIVE TO DEHYDRATION 1 (ERD1)	Leaf senescence associated gene	[40], [111], [112]
GRMZM2G164715	AT5G51640	YELLOW-LEAF-SPECIFIC GENE 7, YLS7	Leaf-senescence-related protein	[7], [82]
GRMZM2G165961	AT5G51640	TRICHOME BIREFRINGENCE-LIKE 17 (TBL17), YELLOW-LEAF-SPECIFIC GENE 7 (YLS7)	leaf-senescence-related protein	[7], [82]
GRMZM2G004183	AT5G51640	TRICHOME BIREFRINGENCE-LIKE 17 (TBL17), YELLOW-LEAF-SPECIFIC GENE 7 (YLS7)	Leaf-senescence-related protein	[7], [82]

There were 33 MYB (MYB-related) and 15 bZIP TF genes with changed expression during leaf senescence (S5 Table), implying that these two large families are involved in maize leaf senescence. This provides an important cue to characterize their function during leaf senescence. Furthermore, 176 of the 236 TFs are found in the plant TFs database described by Zhang et al. (2011) [17] and Pérez-Rodríguez et al. (2010) [16]. The other 60 TFs were considered new transcription factor family members. Their function in maize leaf senescence will be required to be investigated by genetic and molecular analysis.

### GO analysis of genes differentially expressed during natural leaf senescence

Although the functional classification of differentially expressed genes reveals their numbers in each category, the roles of some small gene families in specific biological processes are often ignored. Gene ontology reveals the distribution of every class of gene, both in target samples and in the whole genome, and yields the ontology of defined terms, including the biological process, cellular component, and molecular function, enriched in the target samples using statistical methods. We performed GO analysis on the data using the agriGO toolkit developed by Du et al. (2010) [64](S6 Table).

**Figure 6:**
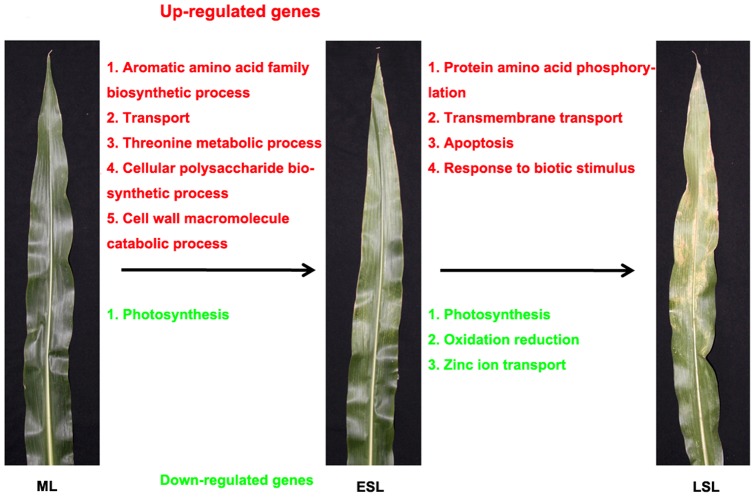
Overrepresented functional GO terms of differentially expressed genes in leaf senescence. Using FDR <0.05 as a criterion, the overrepresented GO terms (biological process) were picked out using the agriGO analysis tool.

The GO analysis revealed that the enrichment of genes differentially expressed during maize leaf senescence focused on 12 biological pathways (Fig. 6). At the early stage of leaf senescence, five pathways including aromatic amino acids (AAAs) biosynthetic process and transport, threonine metabolic process, cellular polysaccharide biosynthetic process, and the cell wall macromolecule catabolic process, were enriched in the up-regulated genes cluster, whereas only one pathway, photosynthesis, was enriched in the down-regulated genes cluster. However, the enriched biological pathways at the late stage were different from those at the early stage. Four pathways, including protein amino acid phosphorylation, transmembrane transport, apoptosis, and response to stimulus were enriched in the up-regulated gene cluster. In addition to photosynthesis, oxidation-reduction and zinc ion transport were enriched in the down-regulated genes cluster. These results show the plant cells not only underwent cellular components and metabolism changes but also would take some protective steps to respond to those changes at the early stage of leaf senescence. Whereas, some processes, e.g. nutrient recycling and cell death, mainly occurred at the late stage. This indicates that there are some differences in molecular mechanisms between these stages during leaf senescence, thus implying the complexity of the senescence process.

### Genes involved in AAAs biosynthesis and metabolism might be involved in the regulation of natural leaf senescence

Amino acids with aromatic rings are characterized as AAAs, e.g., L-trytophan, L-phenylalanine, and L-tyrosine. In plants, AAAs is required for the synthesis of proteins and also for precursors of a diverse range of important natural products [65]. Despite the well-known aspects of AAAs in plant growth, such as reproduction, defense, and environmental response, the roles of AAAs in leaf senescence are poorly understood. As mentioned above, genes involved in AAAs biosynthesis were enriched in the early stage of maize leaf senescence. Subcellular localization of the orthologs of these genes in *Arabidopsis* occurs in chloroplasts (Table 3), consistent with the fact that plastids contain a full set of biosynthetic enzymes for the production of all AAAs [65]. The content of amino acids increased during leaf senescence, which is considered to may have a role in the initiation of the senescence programme [66]. It was also reported that AAAs are important in the normal process of flower senescence [67]. Thus, the biosynthesis and metabolism of AAAs might play a role in the regulation of leaf senescence through catabolic processes in the chloroplasts, although the detailed experimental data need to be further provided in maize.

**Table 3:** The up-regulated genes involved in aromatic amino acid family biosynthetic process in early stage of maize leaf senescence.

Gene ID	Log_2_^(ESL/ML)^	Log_2_^(LSL/ML)^	Orthologous in *Arabidopsis*	Gene symbol	Description	Cellular component
GRMZM2G359822	1.27	1.51	AT4G33510	*DHS2*	phospho-2-dehydro-3-deoxyheptonate aldolase 2	chloroplast, thylakoid
GRMZM2G161337	1.39	0.84	AT2G29690	ASA2	anthranilate synthase component I-2	chloroplast
GRMZM2G365160	2.04	2.13	AT1G22410	\	class-II DAHP synthetase-like protein	chloroplast
GRMZM2G454719	1.52	0.96	AT1G22410	\	class-II DAHP synthetase-like protein	chloroplast
GRMZM2G365961	2.51	2.33	AT1G15710	\	prephenate dehydrogenase family protein	chloroplast
GRMZM2G164562	1.44	1.99	AT1G48850	*EMB1144*	chorismate synthase	chloroplast, stroma
GRMZM2G115841	2.17	1.41	AT2G27820	*PD1*	arogenate dehydratase 3	chloroplast
GRMZM2G003109	2.09	−0.39	AT5G05730	ASA1	anthranilate synthase component I-1	chloroplast, stroma
GRMZM2G051219	2.4	2.7	AT5G17990	*TRP1*	anthranilate phosphoribosyltransferase	chloroplast
GRMZM2G314652	0.13	2.64	AT3G06350	MEE32	NADP or NADPH binding, 3-dehydroquinate dehydratase activity, shikimate 5-dehydrogenase activity, binding, catalytic activity;	chloroplast stroma, chloroplast;
GRMZM2G107639	0	3.05	AT1G80360	\	transferase activity, transferring nitrogenous groups, pyridoxal phosphate binding, catalytic activity;	25 plant structures
GRMZM2G026131	1.82	2.09	AT1G80360	\	transferase activity, transferring nitrogenous groups, pyridoxal phosphate binding, catalytic activity;	25 plant structures
GRMZM2G466534	0	2.74	AT1G08250	ADT6	arogenate dehydratase activity, prephenate dehydratase activity	chloroplast
GRMZM2G123122	1.29	1.77	AT1G22610	\	molecular_function unknown	chloroplast
GRMZM2G014376	1.92	1.45	AT3G06350	MEE32	NADP or NADPH binding, 3-dehydroquinate dehydratase activity, shikimate 5-dehydrogenase activity, binding, catalytic activity;	chloroplast stroma, chloroplast
GRMZM2G029135	1.41	1.06	AT1G80360	\	transferase activity, transferring nitrogenous groups, pyridoxal phosphate binding, catalytic activity;	25 plant structures
GRMZM2G138382	2.94	0.79	AT2G29690	ASA2	anthranilate synthase activity;	chloroplast, anthranilate synthase complex
GRMZM2G171383	1.37	0.2	AT1G25220	ASB1	tryptophan biosynthetic process, response to ethylene stimulus, response to bacterium, auxin biosynthetic process, lateral root primordium development	chloroplast

**Figure 7:**
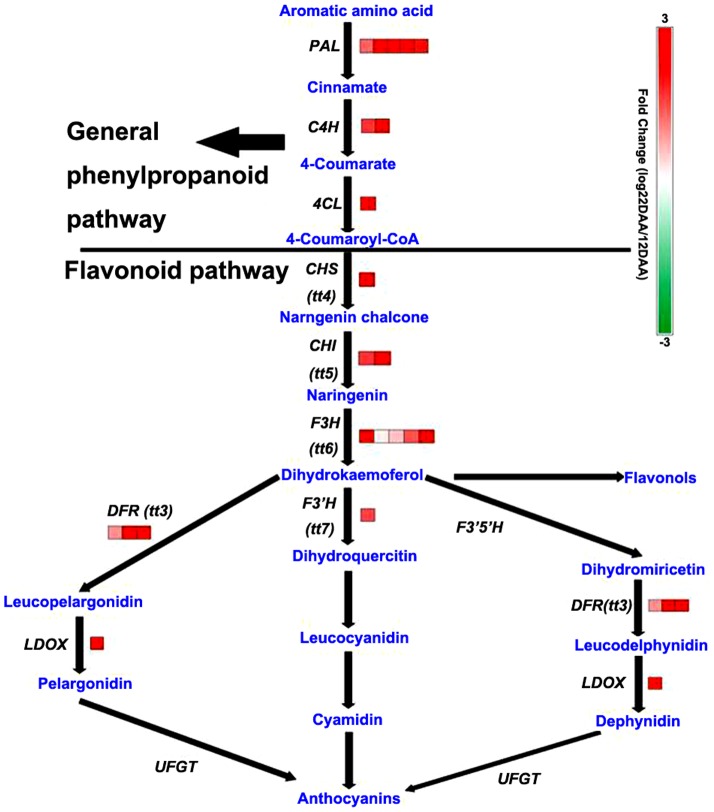
Expression changes of genes involved in flavonoid biosynthesis during maize leaf senescence.

AAAs is precursors for the biosynthesis of flavonoids and phenylpropanoids that increase significantly during the senescence process [68], [69]. Flavonoids in plants have diverse biological functions and are involved in defense against stresses, signalling during nodulation, and pollen fertility [70]. Anthocyanins are a subgroup of flavonoids responsible for the leaf color transition observed in autumn in many plant species. This process is important for the protection of leaf cells against light damage during senescence [71]. It is generally considered that anthocyanins accumulate extensively during the late stage of leaf senescence. However, it was observed that the expression of genes encoding proteins for the biosynthesis of flavonoids and phenylpropanoids, including phenylalanine ammonia-lyase (PAL), cinnamate 4-hydroxylase (C4H), 4-coumaroyl:CoA ligase (4CL), chalcone synthase (CHS), chalcone isomerase (CHI), flavanone 3′-hydroxylase (F3′H), chalcone isomerase (CHI), dihydroflavonol 4-reductase (DFR), and leucoanthocyanidin dioxygenase (LDOX), increased in the early stage of leaf senescence (Fig. 7; S7 Table). This result suggests that anthocyanins were produced during the early stage of leaf senescence, which may attenuate the quality and quantity of light captured and protect the leaf from strong light damage.

### Genes encoding transporters might be required for nutrient remobilization during natural leaf senescence

**Figure 8:**
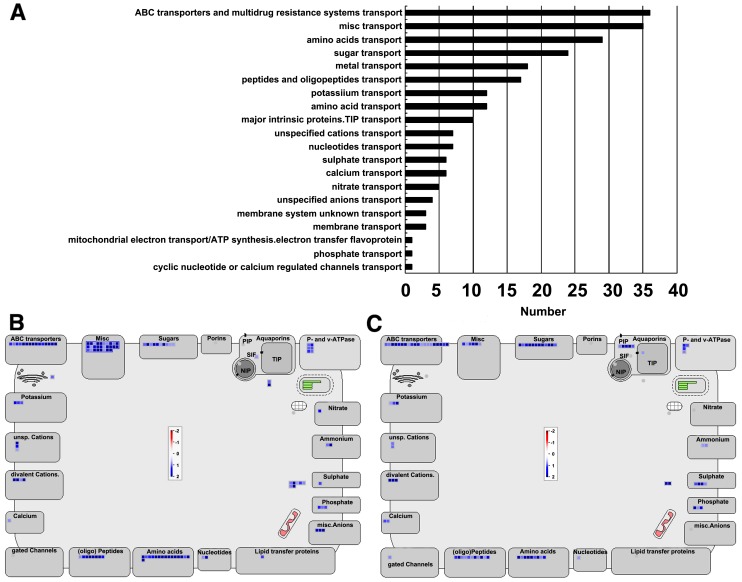
Distribution of transport-related genes in SAGs in maize. (A) Distribution of transport-related genes in maize SAGs. (B) Distribution of the up-regulation expressed genes involved in transport process in early stage of maize leaf senescence. (C) Distribution of the up-regulation expressed genes involved in transport process in advanced stage of maize leaf senescence. The number of genes in each gene family was equal to the number of boxes in each gene family. The relative expression is represented by color scales as indicated.

In many monocotyledon plant species, fruit set and maturation are directly associated with whole-plant senescence. Leaf senescence is essential for the productivity of annual crops such as maize, rice, and wheat because it affects nutrient remobilization efficiency [1], [72]. Transporters play a crucial role in macromolecule degradation and nutrient recycling during plant senescence. Many genes encoding transporters, such as ABC transporters, amino acid permease, and cation exchangers, are senescence-associated, which is consistent with the substantial nutrient translocation during leaf senescence [5]–[7], [73]–[75]. In this study, the expression patterns of 263 genes predicted to encode transporters changed during leaf senescence, and GO analysis revealed that the biological process of transport was enriched in both early and late stage leaf senescence (Fig. 6). These transporter proteins were categorized into 20 classes (Fig. 8A). The transport-related genes that were up-regulated at an early stage of leaf senescence potentially take part in enzyme and amino acid transport (Fig. 8B), suggesting that active metabolism occurs at the beginning of leaf senescence. In contrast, the expression of genes encoding ABC and sugar transporters increased significantly in the late stage of leaf senescence(Fig. 8C), indicating that these genes may play important roles in nutrient recycling at this stage.

### Comparison of genes differentially expressed in natural leaf senescence between maize and *Arabidopsis*

To elucidate the molecular mechanisms underlying leaf senescence, SAGs have been identified in *Arabidopsis* during natural and induced senescence at the genome-wide level [5]–[8], [73], [76]. Breeze and collaborators performed a high-resolution time-course profiling of transcripts during *Arabidopsis* leaf senescence by microarray analysis [8]. To analyze the extent of the similarity of the molecular mechanisms underlying natural leaf senescence between maize and *Arabidopsis*, we performed a BLAST of the amino acid sequences of 4,552 genes differentially expressed during maize leaf senescence in the *Arabidopsis* database [54] using a criterion e-value <1e-10. The comparison results revealed that the 4,552 genes in maize hit 3,935 genes in *Arabidopsis*. These 3,935 genes were compared with the differentially expressed genes described by Breeze et al. (2011) [8]. There were 1,107 genes occupying approximately 28.13% of the total altered expression genes in maize leaf senescence conserved in both SAG datasets (S8 Table). These conserved genes are significantly enriched for genes linked to the chloroplast, thylakoid, and membrane, with function in metabolic processes, particularly photosynthesis, transport, the carboxylic acid catabolic process, the aromatic amino acid catabolic process, lipid metabolism, protein degradation, and hormone metabolism (Table 4, S8 Table), implying that they are conserved in the leaf senescence process of both maize and *Arabidopsis*. Among these, a number of differentially expressed genes in our dataset showed high similarities to the established SAGs identified in *Arabidopsis* (Table 2). These genes and their *Arabidopsis* orthologs probably function in the regulation of leaf senescence in similar ways.

**Table 4:** GO analysis of differentially expressed genes during natural leaf senescence in maize and Arabidopsis.

GO Term^a^	Term type^a^	Query item^b^	Query total^c^	P value^d^	FDR^e^
P	carbohydrate biosynthetic process	30	1097	4.60E-09	5.90E-07
P	carbohydrate metabolic process	64	1097	8.00E-11	1.40E-08
P	carboxylic acid biosynthetic process	34	1097	2.80E-07	2.00E-05
P	carboxylic acid catabolic process	14	1097	3.50E-06	0.00019
P	carboxylic acid metabolic process	61	1097	9.40E-10	1.40E-07
P	cellular carbohydrate biosynthetic process	24	1097	3.20E-09	4.30E-07
P	cellular carbohydrate metabolic process	49	1097	3.80E-15	1.50E-12
P	cellular glucan metabolic process	15	1097	1.80E-07	1.50E-05
P	cellular ketone metabolic process	62	1097	9.90E-10	1.40E-07
P	cellular lipid metabolic process	39	1097	1.40E-05	0.00072
P	cellular metabolic process	359	1097	5.20E-13	1.10E-10
P	cellular nitrogen compound metabolic process	39	1097	1.50E-07	1.30E-05
P	cellular polysaccharide biosynthetic process	15	1097	3.50E-07	2.40E-05
P	cellular polysaccharide metabolic process	18	1097	1.60E-07	1.30E-05
P	cellular process	454	1097	4.00E-13	9.70E-11
P	cellulose biosynthetic process	6	1097	1.90E-05	0.00097
P	establishment of localization	97	1097	5.00E-08	5.30E-06
P	fatty acid metabolic process	23	1097	7.20E-07	4.40E-05
P	glucan biosynthetic process	12	1097	2.90E-07	2.10E-05
P	glucan metabolic process	15	1097	2.40E-07	1.90E-05
P	lipid catabolic process	12	1097	1.90E-05	0.00097
P	lipid metabolic process	51	1097	2.00E-06	0.00012
P	localization	99	1097	7.90E-08	7.80E-06
P	macromolecule modification	101	1097	6.60E-12	1.30E-09
P	metabolic process	447	1097	5.90E-19	5.20E-16
P	monocarboxylic acid metabolic process	36	1097	2.00E-08	2.30E-06
P	organic acid biosynthetic process	34	1097	2.80E-07	2.00E-05
P	organic acid catabolic process	14	1097	3.50E-06	0.00019
P	organic acid metabolic process	61	1097	9.80E-10	1.40E-07
P	oxoacid metabolic process	61	1097	9.40E-10	1.40E-07
P	phosphate metabolic process	98	1097	4.20E-19	5.20E-16
P	phosphorus metabolic process	98	1097	4.40E-19	5.20E-16
P	phosphorylation	88	1097	9.10E-17	4.80E-14
P	polysaccharide biosynthetic process	17	1097	1.50E-07	1.30E-05
P	polysaccharide metabolic process	20	1097	1.00E-07	9.20E-06
P	post-embryonic development	47	1097	4.40E-07	2.90E-05
P	post-translational protein modification	92	1097	5.50E-15	1.80E-12
P	primary metabolic process	370	1097	1.90E-13	5.10E-11
P	protein amino acid phosphorylation	80	1097	3.80E-16	1.70E-13
P	protein modification process	101	1097	1.80E-14	5.40E-12
P	response to abiotic stimulus	93	1097	1.40E-11	2.70E-09
P	response to chemical stimulus	109	1097	7.90E-09	9.60E-07
P	response to endogenous stimulus	58	1097	9.90E-06	0.00054
P	response to external stimulus	34	1097	5.10E-07	3.20E-05
P	response to hormone stimulus	55	1097	7.40E-06	0.00041
P	response to light stimulus	42	1097	4.70E-07	3.00E-05
P	response to organic substance	71	1097	2.40E-06	0.00014
P	response to radiation	43	1097	3.90E-07	2.60E-05
P	response to stimulus	213	1097	6.40E-17	4.20E-14
P	response to stress	115	1097	4.60E-08	5.10E-06
P	secondary metabolic process	39	1097	6.50E-08	6.60E-06
P	transport	96	1097	8.30E-08	7.90E-06

GO terms with *P* value <0.001 and FDR ≤ 0.001 were regarded as overrepresented terms.

a GO term classifications: P, Biological Process.

b The distribution of query genes in each GO term.

c Total annotated query item number in agriGO.

d Determined by Fisher exact test.

e Determined by Benjamini-Hochberg-Yekutieli procedure.

### Comparison of gene expression profile between natural and induced leaf senescence in maize

Leaf senescence is controlled by endogenous factors (e.g., age, ethylene, jasmonic acid, salicylic acid, abscisic acid, and cytokinin) and environmental signals (e.g., drought, darkness, extreme temperature, UV-B irradiation, and pathogen attack) [3]. Transcriptional profiling during senescence induced by the prevention of pollination was investigated in maize by Sekhon et al. (2012) [10]. Comparing our datasets with the ones described by Sekhon et al., it was revealed that only 998 genes overlapped between the two datasets (S9A Table). Some of the major function categories of these genes include the biological processes involved in leaf senescence, e.g. amino acid metabolism, hormone metabolism, lipid metabolism, protein degradation, photosynthesis, transcriptional regulation, and transport. Among the differentially expressed genes, 78.07% of genes during natural maize leaf senescence were not found in the induced senescing maize leaves. Genes specifically expressed in the maize natural senescence process were mainly involved in oxidation reduction, transport, protein amino acid phosphorylation, response to biotic stimulus, the aromatic amino acid family biosynthesis and catabolic process, and the cellular polysaccharide biosynthetic process (Table 5), suggesting that these biological metabolic pathways may play critical roles in the natural senescence of maize.

**Table 5:** Overrepresented GO terms of differentially expressed genes only in the natural senescence.

GO Term^a^	Term type^a^	Query item^b^	Query total^c^	Bg item^d^	Bg total^e^	P value^f^	FDR^g^
oxidation reduction	P	11	201	2006	2743	39203	1.10E-06
transport	P	8	261	2006	3841	39203	1.00E-05
protein amino acid phosphorylation	P	22	166	2006	2387	39203	0.0001
response to biotic stimulus	P	19	8	2006	31	39203	0.00013
aromatic amino acid family biosynthetic process	P	201	11	2006	64	39203	0.00038
aromatic compound catabolic process	P	166	8	2006	37	39203	0.00048
cellular polysaccharide biosynthetic process	P	8	22	2006	195	39203	0.00048
fatty acid biosynthetic process	P	261	19	2006	159	39203	0.00056
photosystem	C	13	13	2006	81	39203	0.00024
electron carrier activity	F	23	85	2006	914	39203	2.10E-07
heme binding	F	85	77	2006	876	39203	5.60E-06
monooxygenase activity	F	77	54	2006	553	39203	7.10E-06
protein serine/threonine kinase activity	F	54	163	2006	2248	39203	1.50E-05
UDP-glucosyltransferase activity	F	5	16	2006	100	39203	5.00E-05
zinc ion transmembrane transporter activity	F	163	5	2006	10	39203	7.20E-05
acyltransferase activity	F	16	23	2006	187	39203	0.0001
oxo-acid-lyase activity	F	5	5	2006	12	39203	0.00021

GO terms with *P* value <0.001 and FDR ≤ 0.05 were regarded as overrepresented terms.

a GO term classifications: P, Biological Process; C, Cellular Component; F, Molecular Function.

b The distribution of query genes in each GO term.

c Total annotated query item number in agriGO.

d Query item number in maize genome version 5a.

e Total annotated item number in maize genome version 5a.

f Determined by Fisher exact test.

g Determined by Benjamini-Hochberg-Yekutieli procedure.

TFs play an important role in leaf senescence. Some TF families involved in leaf senescence, e.g. AP2/EREBP, MYB, WRKY, were found in two datasets. Intriguingly, expression change of *ELF3* (AC233870.1_FG003) was only detected in natural senescence. ELF3 is involved in light deprivation induced leaf senescence and inhibits senescence by repressing PIF4/PIF5 [77]. In addition, 6 genes encoding DNA methyltransferase or histone acetyltransferase show expression level change during natural leaf senescence (S9B Table), implying epigenetic regulation may have a role during natural leaf senescence. From this analysis, it is suggested there are similarities and differences between natural senescence and induced senescence in maize.

## Conclusions

In this study, many genes involved in protein metabolism, transcription, miscellaneous enzyme families, transporters, and signal transduction were identified as differentially expressed during natural leaf senescence in maize. Furthermore, some signalling pathways, such as the biological synthesis of aromatic amino acids, photosynthesis, and transport, were found to play important roles in the regulation of the leaf senescence process in maize. Our data reveal that there are differences in the biological and chemical changes between the early stage and the late stage of leaf senescence. Comparison analyses suggest that 1) the molecular mechanisms of leaf senescence are basically similar between maize and *Arabidopsis* and 2) there are convergence and divergence between natural and induced leaf senescence in maize. These data will extend our understanding of the mechanism of leaf senescence in maize.

## Materials and Methods

### Plant materials and RNA preparation

Plants of maize inbred line Q319 were grown under natural field conditions at Shandong Agricultural University Experimental Station (36°16′ N, 117°16′ E), Tai’an, China during the summer of 2011. The upper parts of the ear leaves (approx. 15–20 cm) (Fig. 1) were harvested at 12, 20, and 28 DAP and were immediately frozen in liquid nitrogen. To reduce biological variation, material was harvested at random from at least three plants and was mixed well.

Total RNA was isolated according to the modified CTAB method described by Gambino et al. (2008)[78] and was purified using an RNeasy MinElute Cleanup Kit (Qiagen, Valencia, CA, USA). The concentration of RNA was measured by Nanodrop spectrophotometry (Nanodrop Technologies, Wilmington, DE, USA), and the RNA quality was examined using an Agilent 2100 Bioanalyzer (Agilent Technologies, Palo Alto, CA, USA). RNA from separate biological samples was used for the construction of libraries.

### Library construction and high-throughput RNA sequencing

Approximately 5 to 8 µg total RNA from each sample was used to construct RNA-Seq libraries. Firstly, the RNA was enriched with poly(A) using oligo(dT) magnetic beads (Illumina, San Diego, CA, USA). Secondly, mRNA was broken into short fragments (approx. 200 bp). Thirdly, first-strand cDNAs were synthesized, using the broken fragments as templates, with random hexamer primers. Then, components including buffer, dNTPs, RNase H, and DNA polymerase I were added to synthesize second-strand cDNA. Next, the cDNA fragments were added with sequencing adaptors. After size-selection by electrophoresis, the required fragments were enriched by PCR amplification. Finally, the quality and quantity of all libraries were assessed by Nanodrop ND-1000 spectroscopy (Thermo Scientific, Waltham, MA) and with an Agilent 2100 Bioanalyzer. All experiments were performed according to the instructions of the RNA-Seq sample preparation kit (Illumina Inc., San Diego, USA; Cat. No. RS-100-0801).

High-throughput next generation transcriptome sequencing using an Illumina HiSeq 2000 was performed on the libraries of leaves at 12, 20, and 28 DAP at the Beijing Genomics Institute (BGI; Shenzhen, China). Original image data were transformed into sequence data via base calling and generated single-end sequence reads. The read quality was evaluated using Illumina Genome Analysis Pipeline (version 1.6) Software. Single-end 49-bp reads were collected. Three types of reads were rejected: (1) reads containing more than 10% unknown bases, (2) reads with an adaptor, and (3) reads containing more than 50% low-quality bases. The remaining reads were defined as filtered clean reads. All sequences are available at the ArrayExpress database (accession number E-MTAB-1709).

### RNA sequencing data analysis

Using the improved Short Oligonucleotide Alignment Program (SOAP2) [43], we mapped filtered clean reads to the AGPv2 maize B73 reference genome [16], [79]. Mismatches of no more than two bases were allowed in the alignment. After the alignment of clean reads, data were divided into three categories: unique matched reads, multi-position matched reads, and unmapped reads. Among these, only unique matched reads were selected for the calculation of the digital gene expression levels. The normalized gene expression levels were calculated as described by Mortazavi et al. (2008) [80] and were reported as reads per kilobase per million reads mapped (RPKM). For one gene having multiple transcripts, the longest transcript was selected to calculate the expression level and coverage.

Pearson correlation coefficients analysis of log2-transformed RPKM values among the RNA-seq libraries were performed using the R package. To reduce sequencing errors, genes with fewer than two clean reads were omitted. The log2-transformed RPKM values of genes expressed in at least one of the three samples were used for PCCs analysis. All log2-tranformed RPKM values less than zero were set to zero. Only tests significant at *p* = 0.05 are shown. The heat map showing the correlation values of the three tissues was drawn by Scalable Vector Graphics.

To identify differentially expressed genes during leaf senescence, the significance of digital gene expression analysis was tested [44]. Using fold change ≥2 and FDR ≤1E-10 as the criteria, genes differentially expressed in at least one of the three comparisons (ESL vs. ML, LSL vs. ML, and LSL vs. ESL) were regarded as differentially expressed during pollination. Gene annotations were derived from AGP v2 5b.60 [42]. Differentially expressed genes were classified into various categories in accordance to MapMan annotation. GO analysis was performed using the Singular Enrichment Analysis tool [81].

Two statistical analyses were conducted to identify overlaps between our SDEG dataset and SAGs identified by Breeze et al. (2011) [8] in *Arabidopsis*. First, the protein sequences of the 3,952 differentially expressed genes were extracted and used as queries to BLAST search against the TAIR 10 *Arabidopsis* protein database [54]. Using E-values ≤1e-10 as the cut-off, the best hit *Arabidopsis* gene was considered as the homolog of each maize gene. Second, homologous genes with identical locus names to those identified by Breeze et al. (2011) [8] were selected. Furthermore, protein sequences of well-known SAGs were extracted and used to BLAST against the maize AGPv2 5b.60-filtered gene set peptide database [42] to find candidate functional genes in maize natural leaf senescence.

### RT-qPCR analysis

Total RNA was extracted using the method described above and then treated with RNase-free DNase I (Promega, Madison, WI, USA) to eliminate genomic DNA. In accordance with the manufacturer's instructions, 4 mg total RNA was used for cDNA synthesis with oligo (dT) primers and M-MLV reverse transcriptase (Promega, Madison, WI, USA). RT-qPCR was performed using SYBR Green Real-time PCR Master Mix (Toyobo, Osaka, Japan) on a CFX96 Real-Time PCR Detection System (Bio-Rad Laboratories Inc., USA). For each gene detected by RT-qPCR, three biological replicates were analyzed. 18S rRNA was used to normalize mRNA levels. Quantitative variations in different replicates were calculated using the delta-delta threshold cycle relative quantification method. The primers used for RT-qPCR are listed in S4 Table.

## Supporting Information

S1 Table **Distribution of reads sequenced from maize natural senescence leaves.** (A) Summary of reads mapped to the reference genome. (B) Summary of reads mapped to the reference gene database.
(DOC)

S2 Table **Genes expressed in maize leaves at three different stages of natural senescence.**
(XLS)

S3 Table **Genes differentially expressed in maize natural leaf senescence.** (A) Genes differentially expressed in the ESL/ML comparison. (B) Genes differentially expressed in the LSL/ML comparison. (C) Genes differentially expressed in the LSL/ESL comparison.
(XLS)

S4 Table **Primers used for RT-qPCR.**
(XLS)

S5 Table **Differentially expressed transcription factors in maize leaves during natural senescence.**
(XLS)

S6 Table **GO analysis of differentially expressed genes encoding transcriptional factors during natural maize leaf senescence.** Using FDR < 0.05 as the criterion, overrepresented GO terms (biological process) were selected using the agriGO analysis tool.^ a^GO term classifications: P, Biological Process; C, Cellular Component; F, Molecular Function. ^b^Query item number in MS preferential expressed genes. ^c^Total annotated query item number in agriGO. ^d^Query item number in maize genome version 5a. ^e^Total annotated item number in maize genome version 5a. ^f^Determined by Fisher exact test. ^g^Determined by Benjamini-Hochberg-Yekutieli procedure.
(XLS)

S7 Table **Expression patterns of differentially expressed genes involved in flavonoid biosynthesis during maize leaf senescence.**
(XLS)

S8 Table **The differentially expressed genes during natural leaf senescence in maize and** ***Arabidopsis*** **.** (A) The overlap of differentially expressed genes during natural leaf senescence in both maize and Arabidopsis. (B) The differentially expressed genes during natural leaf senescence only in maize.
(XLS)

S9 Table **The differentially expressed genes in natural and induced senescence leaves in maize.** (A) The overlap of differentially expressed genes in natural senescence leaf and induced senescence leaf in maize. (B) The differentially expressed genes only in natural senescence leaf in maize. (C) The differentially expressed genes in induced senescence leaf in maize.
(XLS)
