# Supplementary material for: Transcriptional Analyses of Natural Leaf Senescence in Maize
Source: PLoS One. 2014 Dec 22;9(12):e115617. doi: 10.1371/journal.pone.0115617 (PMC4274115; doi:10.1371/journal.pone.0115617)
Supplement: S1 Table — Distribution of reads sequenced from maize natural senescence leaves. (A) Summary of reads mapped to the reference genome. (B) Summary of reads mapped to the reference gene database. (DOC) [file pone.0115617.s001.doc]

**Table S1. Distribution of reads sequenced from maize natural senescence leaves in maize reference genome and reference gene database.** (A) Summary of reads mapped to reference genome. (B) Summary of reads mapped to reference gene database.

(A)

|  | ML | |  | ESL | |  | LSL | |  |
| --- | --- | --- | --- | --- | --- | --- | --- | --- | --- |
|  | reads number | percentage |  | reads number | percentage |  | reads number | percentage |  |
| Total Reads | 11914022 | 100.00% |  | 12381189 | 100.00% |  | 11788942 | 100.00% |  |
| Total BasePairs | 583787078 | 100.00% |  | 606678261 | 100.00% |  | 577658158 | 100.00% |  |
| Total Mapped Reads | 9453880 | 79.35% |  | 9776102 | 78.96% |  | 9081556 | 77.03% |  |
| perfect match | 6738949 | 56.56% |  | 6918968 | 55.88% |  | 6543612 | 55.51% |  |
| <=3bp mismatch | 2714931 | 22.79% |  | 2857134 | 23.08% |  | 2537944 | 21.53% |  |
| unique match | 7493960 | 62.90% |  | 7952689 | 64.23% |  | 7433071 | 63.05% |  |
| multi-position match | 1959920 | 16.45% |  | 1823413 | 14.73% |  | 1648485 | 13.98% |  |
| Total Unmapped Reads | 2460142 | 20.65% |  | 2605087 | 21.04% |  | 2707386 | 22.97% |  |

(B)

|  | ML | |  | ESL | |  | LSL | |  |
| --- | --- | --- | --- | --- | --- | --- | --- | --- | --- |
|  | reads number | percentage |  | reads number | percentage |  | reads number | percentage |  |
| Total Reads | 11914022 | 100.00% |  | 12381189 | 100.00% |  | 11788942 | 100.00% |  |
| Total BasePairs | 583787078 | 100.00% |  | 606678261 | 100.00% |  | 577658158 | 100.00% |  |
| Total Mapped Reads | 8671856 | 72.79% |  | 9430291 | 76.17% |  | 8483666 | 71.96% |  |
| perfect match | 6166050 | 51.75% |  | 6704415 | 54.15% |  | 6130915 | 52.01% |  |
| <=2bp mismatch | 2505806 | 21.03% |  | 2725876 | 22.02% |  | 2352751 | 19.96% |  |
| unique match | 3555047 | 29.84% |  | 3927319 | 31.72% |  | 3718619 | 31.54% |  |
| multi-position match | 5116809 | 42.95% |  | 5502972 | 44.45% |  | 4765047 | 40.42% |  |
| Total Unmapped Reads | 3242166 | 27.21% |  | 2950898 | 23.83% |  | 3305276 | 28.04% |  |
